# Significance of intracranial gas on post-mortem computed tomography in traumatic cases in the context of medico-legal opinions

**DOI:** 10.1007/s12024-019-00162-x

**Published:** 2019-08-28

**Authors:** Aleksandra Borowska-Solonynko, Kacper Koczyk, Katarzyna Blacha, Victoria Prokopowicz

**Affiliations:** 1 Department of Forensic Medicine, Medical University of Warsaw, 1 Oczki st., 02-007 Warsaw, Poland; 2 Forensic Medicine Student Scientific Group, Department of Forensic Medicine, Medical University of Warsaw, 1 Oczki st., 02-007 Warsaw, Poland

**Keywords:** Postmortem computed tomography, Intracranial gas, Pneumocephalus, Intravascular gas, Head injury

## Abstract

The detection of intracranial gas (ICG) in people who died due to trauma became possible once postmortem computed tomography (PMCT) became available in addition to traditional post-mortem examinations. The aim of this study was to determine the importance of ICG in the context of medico-legal opinions. We assessed 159 cases of trauma-induced death. Cadavers with pronounced signs of decomposition, open skull fractures, and after neurosurgical operations were excluded. Both PMCT findings and data from autopsy reports were analyzed. ICG was found in 38.99% (*n* = 62) of the cadavers, 96.77% (*n* = 60) of which presented with pneumocephalus (PNC) and 40.23% (*n* = 25) with intravascular gas (IVG). There was a strong correlation between ICG and skull fractures/brain injuries, as well as chest injuries, especially lung injuries. In 13 cases, ICG presented without skull fractures; three of these cases died as a result of stab and incised wounds to the neck and chest. The mean time between trauma and death was significantly longer in the non-ICG group than the ICG group at 2.94 days (0–48 days) and 0.01 day (0–1 day), respectively (*p* < 0.0001). The presence of ICG is a result of severe neck and chest injuries, including stab and incised wounds. The victims die in a very short amount of time after suffering trauma resulting in ICG. The ability to demonstrate ICG on PMCT scans can be of significance in forming medico-legal opinions.

## Introduction

Traditional autopsy is the primary method used by forensic pathologists for examination of a decedent, but it is prone to certain limitations and occasionally fails to reveal important details [1]. With the rapid advancement of postmortem computed tomography (PMCT), forensic pathologists have gained a powerful new method to complement traditional autopsy [2]. Regardless of the autopsy technique used and the experience of the person conducting the autopsy, it is practically impossible to assess the accumulation of gas in certain parts of the body – including, but not limited to, the skull. Accumulated gas can be reliably detected only via imaging studies [3, 4]. For the above reasons, medico-legal opinions based solely on traditional autopsies disregard the possible presence of intracranial gas (ICG). However, with the popularization of PMCT, ICG have been increasingly recognized. The caveat of adding this finding to a forensic opinion is that the significance of this pathology in the medico-legal context must be known. According to our knowledge, there have been no publications so far that have dealt with this specific issue. Numerous clinical studies on detecting ICG in living patients indicate that this finding may be significant in determining the mechanism and circumstances of death [5–15]. Due to the fact that ICG is mainly observed in cases of traumatic deaths [16], the goal of this study was to determine the frequency and importance of ICG in this group of cadavers in the context of medico-legal opinions.

## Material and methods

The study was conducted retrospectively on PMCT scans acquired in the period between Nov 2014 and Nov 2016. PMCT scans were obtained with a 16-row Astelion CT scanner (Toshiba). In each case unenhanced CT scans were performed, with 1 mm thick slices acquired at 120 V with automatic exposure control (AEC). The pitch factor was 1.438 for the trunk and 0.688 for the head. Cadavers were scanned in a supine position, with the use of the standard protocol, including acquiring scans of the head and neck, the torso, and the lower limbs (if needed). The examination was performed without opening the protective plastic bag or altering the position of the body inside. The PMCT scans were analyzed using OsiriX application (OsiriX MD v.0.8.1, Pixmeo SARL- 266 Rue Bernex, Switzerland). The assessment was conducted independently from the traditional autopsy. Each scan was assessed by a board-certified forensic pathologist with experience in forensic radiology. Ambiguous images were consulted with a board-certified radiologist. The results were reported in a standardized questionnaire.

**Fig. 1:**
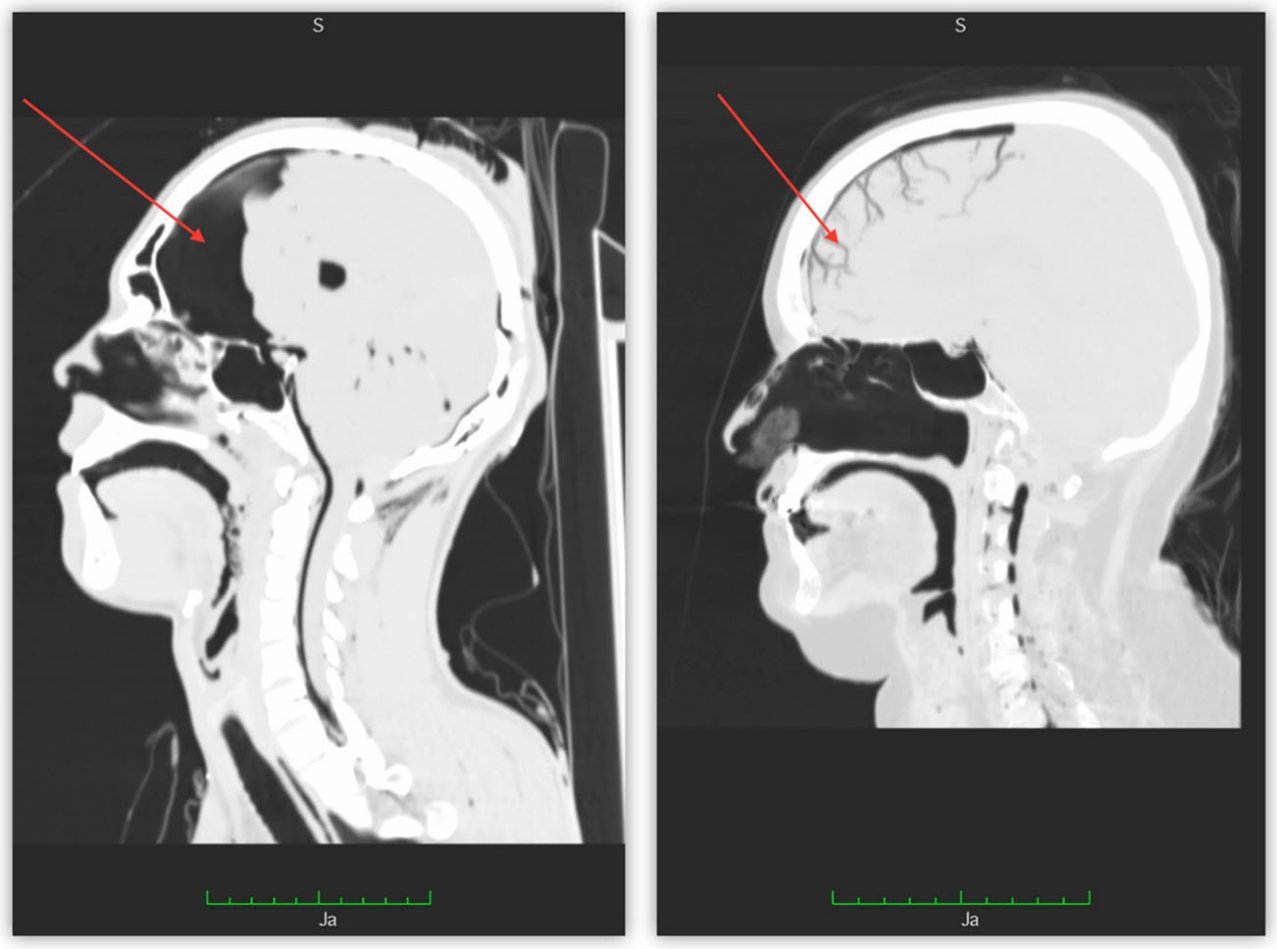
Postmortem computed tomography (PMCT) multiplanar reconstruction (MPR) images showing (**a**) pneumocephalus (PNC) (arrow), **b** intravascular gas (IVG) (arrow)

**Fig. 2:**
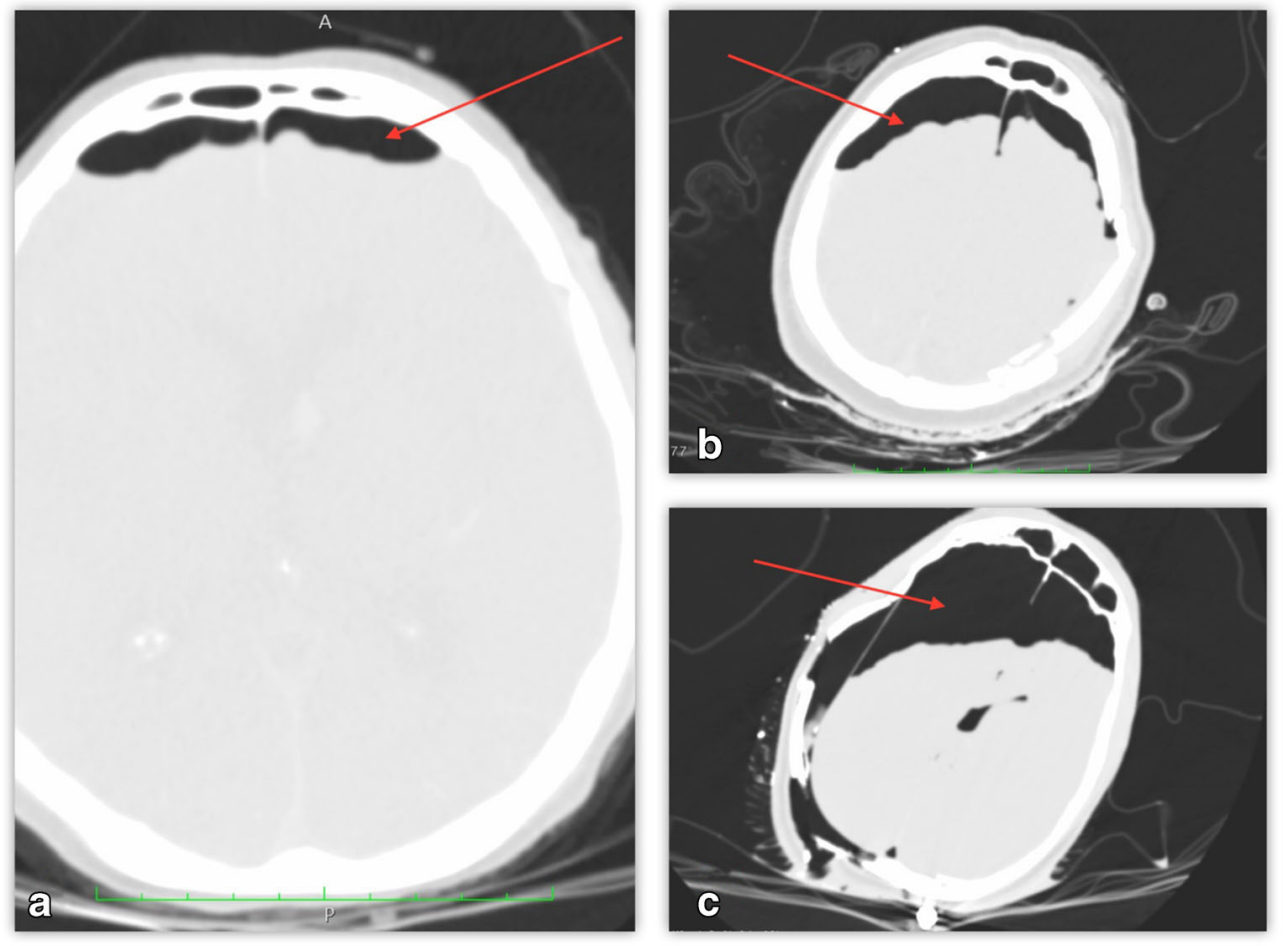
Postmortem computed tomography (PMCT) multiplanar reconstruction (MPR) images showing semi-quantitative analysis of pneumocephalus (PNC). **a** PNC I, **b** PNC II, **c** PNC III

Initially, a total of 492 scans were evaluated. Ultimately, 159 cases of traumatic deaths were included in the study, after exclusion of cases with evident opening of the skull (excerebrations, open fractures, neurosurgical operations) and cases with extensive putrefaction. The degree of putrefactive development was assessed on the basis of changes described in the autopsy reports, with each pertinent autopsy conducted at least one day after the PMCT examination. Only cases with no, or slight, signs of decomposition, such as green discoloration of the lower abdomen, were included in the study. The mean age of the deceased was 50.4 years (0.01–90 years) and 69.8% (*n* = 111) of all cases were male. The largest number of cases were victims of traffic accidents 52.8% (*n* = 84), followed by fall from height 26.41% (*n* = 42), blunt injury (low force) 11.95% (*n* = 19), stab or cut wounds 7.54% (*n* = 12), and gunshot wounds 1.25% (*n* = 2). The primary data was collected with regard to the presence of ICG, which was categorized into two groups based on its location: PNC, defined as gas present between the cerebral surface and the inner table of the cranium (regardless of the gas volume), and IVG, defined as the gas present inside cerebral arteries or venous sinuses (Fig. 1). There were 22 cases in which both forms of gas collection were present simultaneously. Additionally, PNC was assessed semi-quantitatively based on cross-sectional images of the head in the PMCT and described as small (PNC I – gas visible in the form of a narrow band in the frontal part of the cranial cavity), moderate (PNC II – gas visible in the form of a distinct band covering the anterolateral parts of the cranial cavity) or large (PNC III – gas-filled space occupies much of the anterior part of the cranial cavity and extends to the back of the skull) (Fig. 2). Selective analyses were also performed, with the exclusion of cases in which PMCT results revealed the presence of intrahepatic gas, as in several publications this was an indication of an ongoing putrefactive processes. A total of 93.8% of evaluated cadavers underwent a traditional autopsy performed by an experienced forensic pathologist; in these cases, pertinent data was also obtained from the autopsy reports. The analyzed data included the subject’s age, sex, mechanism of death, cause of death, time from death to PMCT, time from trauma to death (whether or not death took place in a hospital), the time from trauma to PMCT, body position at the time of injury (vertical or not), the presence of gas in other body spaces and organs, and certain organ and bone injuries (selected with regard to the possible impact on the occurrence of ICG.

TIBCO Software Inc. (2017) Statistica (data analysis software system, version 13, Statsoft Polska, ul. Kraszewskiego 36, Kraków) was used for statistical analysis. The chi-square test and Fischer’s exact test were used to compare groups of categorical variables. The influence of continuous data on study groups was assessed using a two-sample t-test for parametric variables and the Mann-Whitney U test for non-parametric variables. The chi-square or Kolmogorov-Smirnov tests were used in order to determine the distribution of data. The phi coefficient (Φ) was used to determine the correlation between data. The results were considered statistically significant when the adjusted *p* values were less than 0.05 (*p* < 0.05).

## Results

### Initial analysis

ICG was found in 38.99% (*n* = 62) of cadavers; out of those 96.77% (*n* = 60) presented with PNC and 40.23% (*n* = 25) with IVG. The mean age of the examined decedents in the ICG vs. non-ICG groups was similar at 50.5 years vs. 52 years (*p* = 0.6), respectively. Similarly, there was no significant difference between the aforementioned groups in regard to sex. In both groups, men were the majority, constituting 69.35% (*n* = 43) of the ICG group and 70.1% (*n* = 68) of the non-ICG group (*p* = 0.92). The median time from death to PMCT was 2 days (0–13 days), both in the entire study group and in the ICG and non-ICG subgroups. Also for all groups, the median time from death to autopsy was 5 days (2–14 days). During autopsy, the onset of decomposition was observed slightly more frequently in the non-ICG group (26.04%, *n* = 25) than in the ICG group (19.23%, *n* = 10); however, this finding was not statistically significant. Analysis showed non-significant differences in the proportion of ICG-positive decedents between the groups of different mechanisms of death (*p* = 0.19) and different causes of death (*p* = 0.28). Those in the ICG group died in the hospital less often than those from the non-ICG group (13.46% vs. 42.7%, *p* = 0.00029). The time between trauma and death was significantly longer in the non-ICG group than that in the ICG group and was on average 2.94 days (0–48 days) and 0.01 day (0–1 day) respectively (*p* < 0.0001).

The position of the body at the time of injury was determined in 49% (*n* = 78) of cases based on the information provided in the prosecutorial order to perform autopsy. There was no statistically significant relationship between the position of the body at the time of the injury and the presence of ICG. Out of 31 cases from the ICG group with known position of the body, 30 victims (96.77%) were recorded to have been standing upright. In the IVG subgroup all victims whose body position was confirmed (*n* = 14) had been in a vertical position. However, the findings in the non-ICG group were similar, with 91.49% (*n* = 43) out of the 47 cases in which the victim’s body position was confirmed also reported to have been standing upright. These figures could be misleading due to the fact that the position of the body could not be determined in the majority of cases, and when it was determined, it was practically universally vertical, with only isolated instances of a supine position.

### The relationship between ICG and the presence of gas in other body spaces and organs

ICG was sometimes accompanied by the presence of gas in other body spaces and organs (Table 1). ICG most frequently co-occurred with gas in the cervical spinal canal, right and left cardiac ventricles, liver, and blood vessels of the neck. These relationships were statistically significant (*p* < 0.0001). The strongest correlations were found between ICG and gas in the spinal canal (Φ =0.68) and between ICG and gas in the vessels of the neck (Φ =0.52). There was also a statistically significant relationship between the presence of IVG and the occurrence of gas in the soft tissues of the neck (*p* = 0.019) and/or the mediastinum (*p* = 0.00095). There were additional statistically significant relationships between PNC and gas in the thoracic spinal canal (*p* = 0.0001) and pneumothorax (*p* = 0.002). A similar analysis was performed after excluding the cases with PMCT-based evidence of gas in the liver – without dividing the ICG groups into PNC and IVG. This analysis showed decreased proportions of ICG coexisting with gas in all other evaluated body spaces. However, similarly to the results of the entire study group, even with exclusion of cases with gas in the liver, there was a statistically significant relationship between ICG and both the presence of gas in the spinal canal and the vessels of the neck (*p* < 0.0001), confirmed by high correlation coefficients of Φ = 0.68 and Φ = 0.48, respectively.

**Table 1:** Presence of gas in other organs in relation to the presence of ICG - the entire study group. Statistically significant results were bolded (due to autopsy not being conducted in some cases, there is missing data regarding some injuries; the percentage values refer only to the cases with known data)

Presence of gas in extracranial locations	ICG group		non–ICG group
	PNC	IVG	
Gas in the cervical vessels	**46.67% (*n*** **= 28)**	**56% (n = 14)**	**2.6% (n = 2)**
Gas in the soft tissue of the neck	20% (n = 12)	**28% (n = 7)**	**7.59% (n = 6)**
Gas in the cervical spinal canal	**61.67% (*n*** **= 37)**	**80% (*n*** **= 20)**	**0**
Gas in the thoracic spinal canal	**17.39% (n = 8)**	31.25% (*n* = 5)	0
Pneumothorax	**63.33% (*n*** **= 38)**	56% (n = 14)	46.39% (*n* = 45)
Gas in the mediastinum	23.21% (*n* = 13)	**40.91% (*n*** **= 9)**	12.37% (n = 12)
Gas in the pericardial sac	26.83% (n = 11)	25% (n = 3)	14.47% (n = 11)
Gas in the right ventricle	**72.88% (n = 43)**	**84% (*n*** **= 21)**	**23.66% (*n*** **= 22)**
Gas in the left ventricle	**33.9% (n = 20)**	**44% (n = 11)**	**11.84% (n = 9)**
Gas in the liver	**64.41% (n = 38)**	**80% (n = 20)**	**27.47% (n = 25)**

### The relationship between ICG and selected soft-tissue and bone injuries

Due to the fact that the examined subjects died as a result of trauma, ICG was found together with numerous injuries (Table 2). The clearest association was found between ICG and skull injuries, this relationship was statistically significant (*p* < 0.0001). However, this correlation was clearly stronger in cases with PNC than in those with IVG (Φ = 0.44 vs. Φ = 0.25). Further statistically significant relationships were found between PNC on one hand and brain injuries (*p* = 0.003), intracranial bleeding (*p* = 0.028), rib fractures (*p* = 0.0033), and lung injuries (*p* = 0.004) on the other. A similar analysis was performed after excluding cases in which PMCT showed gas in the liver; this time without dividing ICG into PNC or IVG (as described in the previous paragraph) (Table 3). As a result of this reduction in the number of analyzed cases we observed a decrease in the percentage of some types of extracranial injuries, whereas the percentage of certain other extracranial injuries increased. The latter injuries were: fractures of the cervical spine, spinal cord injuries, rib fractures, and damage to the aorta. New, statistically significant relationships between ICG and thoracic spine fractures (*p* = 0.027) and between ICG and aortic damage (*p* = 0.034) were observed.

**Table 2:** Other body injuries in relation to the presence of ICG – whole test group. Statistically significant results were bolded (due to autopsy not being conducted in some cases, there is missing data regarding some injuries; the percentage values refer only to the cases with known data)

Other injuries	ICG		non ICG
	PNC	IVG	
Cranial vault fractures	**76.67% (*n*** **= 46)**	**76% (n = 19)**	**29.9% (*n*** **= 29)**
Skull base fractures	**66.67% (*n*** **= 40)**	**68% (*n*** **= 17)**	**25.77% (n = 25)**
Depressed fractures	**20% (n = 10)**	12.5% (n = 3)	**5.21% (n = 5)**
Brain injuries	**84.31% (n = 43)**	83.3% (n = 20)	**60.82% (*n*** **= 59)**
Intracranial bleeding	**78.33% (*n*** **= 47)**	76% (n = 19)	**61.85% (n = 60)**
Fractures of the cervical spine	22.03% (n = 13)	25% (n = 6)	26.8% (*n* = 26)
Fractures of the thoracic spine	42.37% (n = 25)	37.5% (n = 9)	27.84% (*n* = 27)
Spinal cord transection	16.00% (n = 8)	8.33% (n = 2)	9.38% (n = 9)
Sternum fractures	35.59% (n = 21)	33.33% (*n* = 8)	34.02% (*n* = 33)
Rib fractures	**88.33% (*n*** **= 53)**	76% (n = 19)	**69.07% (*n*** **= 67)**
Lung injuries	**84% (n = 42)**	75% (*n* = 18)	**61.05% (*n*** **= 58)**
Heart injuries	13.33% (n = 8)	20% (n = 5)	14.43% (n = 14)
Aorta injuries	34% (n = 17)	20.83% (n = 5)	27.08% (n = 26)
Liver injuries	48% (*n* = 24)	45.83% (n = 11)	40.86% (n = 38)

**Table 3:** Occurrence of bone and soft tissue injuries together with ICG – after exclusion of cases of gas in the liver. Statistically significant results were bolded

Other injuries	ICG
Cranial vault fractures	**59.09% (n = 13)**
Skull base fractures	**59% (n = 13)**
Depressed fractures	16.67% (n = 3)
Brain injuries	66.67% (n = 12)
Intracranial bleeding	68.18% (*n* = 15)
Fractures of the cervical spine	13.64% (n = 3)
Fractures of the thoracic spine	**45.44% (n = 10)**
Spinal cord transection	22.22% (n = 4)
Sternum fractures	40.91% (n = 9)
Rib fractures	**90.91% (n = 20)**
Lung injuries	**83.33% (n = 15)**
Heart injuries	13.64% (n = 3)
Aorta injuries	**50% (n = 9)**
Liver injuries	38.89% (n = 7)

## Analyzing the relationship between the size of PNC and other factors

There were no statistically significant differences between the subgroups characterized by a given extent of PNC with respect to age, time from injury to death, or time from injury to PMCT, even though the time varied in the latter case. The median time between injury and PMCT stratified by PNC size was 4 days for PNC I, 2 days for PNC II, and 1.5 days for PNC III. Only 7 people with PNC died in hospital, all of whom had PNC I or PNC II. There was one case of in-hospital survival of over 24 h (PNC II), in the remaining cases, regardless of the extent of PNC, survival time was limited to a few hours after trauma. The incidence of gas in specific body spaces and organs is presented in Table 4; the frequency of injuries to internal organs and bones in relation to PNC size is presented in Table 5. There was no statistically significant correlation between PNC size and the incidence of gas in extracranial areas of the body. Nevertheless, a direct positive relationship was observed between the size of PNC and the incidence of gas in the liver – with 50% of PNC I cases, 65.71% of PNC II cases, and 80% of PNC III cases showing gas in the liver. No such relationship was observed in regard to other places in the body which were examined for gas accumulation. Only one statistically significant relationship was found in regard to organ and bone injuries – the relationship between PNC grades and brain injuries (*p* = 0.027), with a correlation coefficient Φ = 0.35. Cerebral injuries were observed in all cases of PNC III. Increasing grades of PNC were also found to coexist with increasing numbers of the following injuries: cranial vault fractures, skull bases fractures, and depressed fractures of the skull. All cases of PNC III were accompanied by a fracture of the cranial vault (similarly to the case of brain injuries). Despite this, this relationship was not statistically significant.

**Table 4:** The presence of gas in other organs in relation to pneumocephalus (PNC) size (due to autopsy not being conducted in some cases, there is missing data regarding some injuries; the percentage values refer only to the cases with known data)

	PNC I	PNC II	PNC III
Gas in the cervical vessels	33.33% (n = 5)	54.29% (n = 19)	40% (n = 4)
Gas in the soft tissues of the neck	20% (n = 3)	20% (n = 7)	20% (n = 2)
Gas in the cervical spinal canal	66.67% (n = 10)	57.14% (n = 20)	70% (n = 7)
Gas in the thoracic spinal canal	18.18% (n = 2)	14.81% (n = 4)	25% (n = 2)
Pneumothorax	73.33% (n = 11)	51.43% (*n* = 18)	90% (n = 9)
Gas in the mediastinum	33% (n = 4)	22.86% (n = 8)	11.11% (n = 1)
Gas in the pericardial sac	55.56% (n = 5)	20.83% (n = 5)	12.5% (n = 1)
Gas in the right ventricle	71.43% (n = 10)	68.75% (n = 24)	90% (n = 9)
Gas in the left ventricle	50% (n = 7)	25.71% (n = 4)	40% (n = 4)
Gas in the liver	50% (n = 7)	65.71% (*n* = 23)	80% (n = 8)

**Table 5:** Bone and soft tissue injuries in relation to pneumocephalus (PNC) size. Statistically significant results were bolded (due to autopsy not being conducted in some cases, there is missing data regarding some injuries; the percentage values refer only to the cases with known data)

	PNC I	PNC II	PNC III
Cranial vault fractures	53.33% (n = 8)	80% (n = 28)	100% (n = 10)
Skull base fractures	46.67% (n = 7)	68.75% (n = 24)	90% (n = 9)
Depressed fractures	0%	25% (n = 7)	37.5% (n = 3)
Brain injuries	**64,29% (n = 9)**	**89.66% (n = 26)**	**100% (n = 8)**
Intracranial bleeding	73.33% (n = 11)	82.86% (n = 29)	70% (n = 7)
Fractures of the cervical spine	20% (n = 3)	23.53% (n = 8)	20% (n = 2)
Fractures of the thoracic spine	53.33% (n = 8)	38.24% (n = 13)	40% (n = 4)
Spinal cord transection	14.29% (n = 2)	17.86% (n = 5)	12.5% (n = 8)
Sternum fractures	33.33% (n = 5)	35.29% (n = 12)	40% (n = 4)
Rib fractures	86.67% (n = 13)	88.57% (*n* = 31)	90% (n = 9)
Lung injuries	85.71% (*n* = 12)	52.38% (n = 22)	100% (n = 8)
Heart injuries	20% (n = 3)	8.57% (n = 2)	20% (n = 2)
Aorta injuries	42.86% (n = 6)	32.14% (n = 9)	25% (n = 2)
Liver injuries	50% (n = 7)	42.86% (n = 12)	62.50% (n = 5)

### Cases of ICG without skull fracture

A separate analysis was undertaken of cases in which ICG was not accompanied by any skull fracture. There were 13 such cases in the whole study group, including seven victims of traffic accidents, three cases of falls from heights, and three victims of stab and incised wounds. Those wounds in all three victims were located on the neck and caused damage to the airways; with one victim additionally demonstrating injury to the internal carotid vein and two victims demonstrating chest wounds. It was not possible to determine the position of the victims at the time of stabbing/incision. In one of these cases, not only was there pronounced ICG, but also damage to the aorta, spinal cord, and cervical vertebral column. In the two remaining cases, PNC was barely visible, but gas was visible inside the vessels (IVG). In all three cases, stab and incision wounds were likewise present in the vessels and soft tissues of the neck. None of these cases featured gas in the left cardiac ventricle; however, two cases presented with gas in the right ventricle.

The remaining ten cases were victims of traffic accidents and falls from height. Eight of these ten victims had died instantly, with the remaining two dying in the hospital; all victims died within 24 h from sustaining injuries. PNC was observed in all ten decedents, four of whom, all having died at the scene of the accident, presented with marked PNC (grades II and III) and the remaining six showed a barely visible band of gas (PNC I). Additionally, there was gas in the cerebral vessels (IVG) in four cases. Five of the ten victims had no head injuries, but had either chest injuries alone (in three cases) or injuries to both the chest and abdomen (in the remaining two). Lung damage was observed in eight of the ten victims, aortic damage in seven, gas in the right ventricle in five, and gas in the left ventricle in four. Only in three of the ten victims there was gas in the liver, however, this was accompanied by injuries to the liver. The position of the body at the time of injury could be determined in six of the ten people (all six being victims of traffic accidents), with five of these reported to have been standing upright and only one to have been supine (that last victim was run over by a tram, which resulted in a total transection of the body at the level of the abdomen).

## Discussion

Computed tomography (CT) is an excellent method for diagnosing PNC, as it can help detect as little as 0.5 mL of gas, due to its characteristic very low radiodensity (approx. -1000 HU) [17]. In clinical practice PNC is a commonly diagnosed phenomenon associated with surgical interventions to the skull, spine, or paranasal sinuses. It is especially common as a side effect of operations on the base of the skull [10]. In a study in 131 patients undergoing supratentorial craniotomy, immediately postoperative CT showed PNC in every case [18]. The time of postoperative PNC resolution is not well defined; PNC can still persist up to 3 weeks after surgery [18]. There are also cases of gas emboli in vessels, including cerebral vessels, caused by intravenous puncture [7, 13, 19]. In addition, ICG in the living is observed in barotrauma [19], certain infections, and oncological diseases [20]. However, the main cause of pre-mortem ICG in non-hospital settings is trauma. However, in clinical practice post-traumatic ICG is considered a rare complication, with its reported rates ranging from 0.5% [20] to 9.7% [12] of injury cases (mostly head injuries). There are also published cases of pre-mortem, post-traumatic ICG not associated with heady injury, such as ICG following stab wounds [14]. In medico-legal literature, ICG is only discussed in the context of the mechanism of death by gas embolism which is further accompanied by gas embolism in the cerebral vessels, such as cases of decompression in divers [21], administration of intravenous oxygen [22], or helium intoxication. [23]. Most of the relevant publications mention ICG as one of the pathologies detectable only since the introduction of PMCT [1–4]. The presence of gas in the skull is also discussed together with other gas locations in PMCT results [16]. The phenomenon of gas visualized with PMCT, mainly in non-traumatic cases [24, 25], has been presented in the literature in the context of attempting to differentiate pre-mortem from post-mortem (i.e. related to decomposition) gas accumulations. The results of these studies are not clear-cut. Some authors emphasize the fact that the majority of post-mortem gas is the result of decomposition and should be treated as an artifact [26], even if there is no evidence of ongoing putrefaction [27]. Other authors highlight the fact that their studies did not confirm the decompositional origin of the gas visible in PMCT [28], especially when the CT scan was performed in the first 24 h after death. Due to the difficulties of definitively determining the origin of gas in decedents [29], the conclusions of these studies do not provide a base for using this PMCT-gathered data for creating medico-legal opinions. Our research, covering a large group of people who died in a traumatic mechanism, showed a much more frequent occurrence of ICG in this group of decedents in relation to the frequency of ICG observed in living people following injury. This phenomenon can be explained by the fact that most people with this complication were shown to have died at the scene of the event [30]. There was also a strong correlation between PNC and chest injuries, especially lung injuries. The mechanism in which damaged lungs can cause the entry of gas into the venous and arterial vessels of the brain has been explained in the literature as due to the rupture of both the alveoli and the surrounding small vessels in cases of blunt chest injuries [24, 31] and damage to larger blood vessels of the chest in cases of penetrating injuries [14]. Our study’s most important finding was that most people presenting with ICG died at the site where they suffered their injuries, and even if their vital functions were maintained long enough for hospitalization, death occurred within 24 h. Our study did not draw a clear-cut conclusion in regard to the mechanism in which the presence of ICG affects the patient’s deterioration. However, several hypotheses can be considered. One of them is the possibility that ICG causes an increase in intracranial pressure in the same way that intracranial hematomas do [6, 12, 19, 32]. The patient’s deterioration may also result from gas embolism, which is present in a significant number of PNC cases and affects both intracranial vessels [12, 33] and the vessels of the neck and heart. The results of our research do not support the statement that the chance of the observed ICG being due to decomposition is high enough to reduce the significance of the finding itself. In fact, evidence of early putrefaction was found more often in the non-ICG group than the ICG one. The median time from trauma to PMCT decreased with the observation of larger PNCs. Additional calculations were performed after excluding cases with the presence of gas in the liver, which, according to the authors of some publications, indicates an ongoing process of decomposition, despite the lack of visible signs on the outside of the body [34–37]. Nonetheless, this did not significantly change the relationships found in the entire study group, which indicates that the presence of gas in the liver in the investigated cases may have been a consequence of the injury itself and not of putrefactive processes. This can be confirmed in part by certain publications [16, 38, 39]. The relative increase in the rates of aortic damage in the ICG group after the exclusion of cases with intrahepatic gas may be explained by gas not being able to move to the liver due to sudden interruption of blood circulation. Our study indicates that PMCT-based evidence of ICG in traumatic cases with no open skull fractures and no evident signs of decomposition (either on autopsy or PMCT) should be mentioned in medico-legal opinions as one of the significant consequences of trauma that can severely worsen prognosis. This element of prognosis may be particularly important in opinions regarding the withholding of assistance, such as those involving a driver causing a traffic accident and its victim. In such situations, the medical forensic expert usually has to answer the question whether the nature of injuries indicates that the risk of death would have been significantly lower if assistance had been given within a short time after the injury. Our study failed to prove a relationship between ICG and the body position due to difficulties in obtaining this data. Further research in this area is necessary.

## Limitations

It was impossible to examine the chemical composition of gas observed in PMCT in the center where the research was conducted. Furthermore, due to an insufficient amount of relevant data, we were unable to reliably analyze the relationship between ICG and the possible resuscitation efforts or ICG and the victim’s body position at the time of injury.

## Key points

1. The presence of ICG correlates with severe injuries of the head and lungs.
2. The presence of gas in the cranial cavity is often accompanied by gas in other cavities of the body, especially the spinal canal but also in the heart ventricles and liver.
3. The presence of ICG as a result of trauma determined death on the spot or, at most, after 24 h of hospitalization.
4. The presence of ICG in forensically examined cadavers, which is currently possible to demonstrate through the use of PMCT examinations, may be an important element of medico-legal opinions.
